# An assessment of the malaria-related knowledge and practices of Tanzania’s drug retailers: exploring the impact of drug store accreditation

**DOI:** 10.1186/s12913-018-2966-4

**Published:** 2018-03-09

**Authors:** Rebecca Thomson, Boniface Johanes, Charles Festo, Admirabilis Kalolella, Mark Taylor, Sarah Tougher, Yazoume Ye, Andrea Mann, Ruilin Ren, Katia Bruxvoort, Barbara Willey, Fred Arnold, Kara Hanson, Catherine Goodman

**Affiliations:** 1 London School of Hygiene and Tropical Medicine, London, UK; 2 Ifakara Health Institute, Dar es Salaam, Tanzania; 3 Department of Public Health, Trnava University, Trnava, Slovakia; 4 International Health Division, ICF International, Calverton, MD USA

**Keywords:** Accredited drug dispensing outlets, Private sector drug retailers, Tanzania, Malaria, Antimalarials

## Abstract

**Background:**

Since 2003 Tanzania has upgraded its approximately 7000 drug stores to Accredited Drug Dispensing Outlets (ADDOs), involving dispenser training, introduction of record keeping and enhanced regulation. Prior to accreditation, drug stores could officially stock over-the-counter medicines only, though many stocked prescription-only antimalarials. ADDOs are permitted to stock 49 prescription-only medicines, including artemisinin combination therapies and one form of quinine injectable. Oral artemisinin monotherapies and other injectables were not permitted at any time. By late 2011 conversion was complete in 14 of 21 regions. We explored variation in malaria-related knowledge and practices of drug retailers in ADDO and non-ADDO regions.

**Methods:**

Data were collected as part of the Independent Evaluation of the Affordable Medicines Facility - malaria (AMFm), involving a nationally representative survey of antimalarial retailers in October-December 2011. We randomly selected 49 wards and interviewed all drug stores stocking antimalarials. We compare ADDO and non-ADDO regions, excluding the largest city, Dar es Salaam, due to the unique characteristics of its market.

**Results:**

Interviews were conducted in 133 drug stores in ADDO regions and 119 in non-ADDO regions. Staff qualifications were very similar in both areas. There was no significant difference in the availability of the first line antimalarial (68.9% in ADDO regions and 65.2% in non-ADDO regions); both areas had over 98% availability of non-artemisinin therapies and below 3.0% of artemisinin monotherapies. Staff in ADDO regions had better knowledge of the first line antimalarial than non-ADDO regions (99.5% and 91.5%, *p* = 0.001). There was weak evidence of a lower price and higher market share of the first line antimalarial in ADDO regions. Drug stores in ADDO regions were more likely to stock ADDO-certified injectables than those in non-ADDO regions (23.0% and 3.9%, *p* = 0.005).

**Conclusions:**

ADDO conversion is frequently cited as a model for improving retail sector drug provision. Drug stores in ADDO regions performed better on some indicators, possibly indicating some small benefits from ADDO conversion, but also weaknesses in ADDO regulation and high staff turnover. More evidence is needed on the value-added and value for money of the ADDO roll out to inform retail policy in Tanzania and elsewhere.

## Background

In many developing countries the private sector has played an increasingly important role in treatment seeking in recent years, accounting for 40-60% of care-seeking visits [1]. Treatment for common illnesses is often obtained in drug shops, as there are frequent stock-outs of essential drugs in the public sector, while drug stores are often more geographically accessible than public facilities, have longer opening hours, and better stock availability [2]. However, there are concerns that drug stores are poorly monitored and may be run by under-qualified staff [3]. Working with such private medicine sellers to improve their quality of care has therefore been seen as a strategy to enhance overall health service provision [4]. Strategies that have been used include training of drug retailer staff, social marketing and social franchising [5–7].

In Tanzania the private drug retail sector has been expanding rapidly. A study in three districts found that the number of drug stores had doubled between 2004 and 2008 [2], while a study in three regions found that 54% of people with fever visited a drug store or pharmacy for their first source of care in 2012, up from 41% in 2010 [8]. Until 2003, the two types of retail outlets permitted to sell drugs in Tanzania were pharmacies and drug stores, the latter termed ‘duka la dawa baridi’ (DLDB) in Kiswahili. Tanzania has only around 700 pharmacies and they are mainly found in urban areas, so the 7000-plus drug stores have been a crucial source of care in rural and peri-urban areas [9, 10]. Drug store staff were required to have at least 4 years of health-related training, and were officially allowed to stock over-the-counter medication (OTC) only, but often also sold prescription only medicines (POMs) [11]. Further concerns about their operations related to evidence of inadequate facilities for storing medications, under-qualified staff, and the limited supervision received from inspectors [9, 11]. A small proportion of general retailers also stocked OTCs, although they were not permitted to do so [12].

Since 2003 the Tanzanian Government, with support from Management Sciences for Health, has aimed to improve healthcare provision in the retail sector by upgrading drug stores to Accredited Drug Dispensing Outlets (ADDOs), with the aim of increasing access to certain POMs and to good quality pharmaceutical services, especially in rural areas where there are few registered pharmacies [9]. Upgrading involves a 35-day dispenser training for outlet staff, covering a number of medical issues such as dispensing antimalarials, Integrated Management of Childhood Illnesses (IMCI), family planning and HIV/AIDS care, as well as introduction of record keeping [13]. Regulation is also supposed to be enhanced through monitoring and evaluation by ward and district officials to strengthen local regulatory capacity [14]. ADDOs are permitted to stock a specified list of 49 POMs. Non-ADDO drug stores are officially not permitted to exist in ADDO regions.

The Ministry of Health and Social Welfare decided to roll out the ADDO programme nationwide starting in 2006. Roll out occurred by region, with the intention of covering the more rural areas first [9]. By the end of 2010, eight of Tanzania’s then 21 regions had been upgraded, with a further six completed in early 2011, and all remaining regions covered by 2013 [15]. In addition, a one-day retraining programme covering malaria, including how to dispense ACTs, IMCI and family planning was conducted in two regions in August and September 2011.

While there is a substantial literature on the ADDO programme [2, 3, 9, 10, 16–19], few studies have aimed to evaluate drug store accreditation by presenting comparable data on ADDO and non-ADDO drug stores. A few studies have compared ADDOs with other types of provider such as public health facilities [20, 21] or pharmacies [22], but only two studies compared ADDOs with non-ADDOs, only one of which focused on malaria treatment [23, 24]. A pilot of the ADDO programme was evaluated by the ADDO implementing team in 2003-2004 by comparing ADDOs in four districts of Ruvuma region in southern Tanzania with DLDB in four comparison districts in Singida region in central Tanzania, documenting mixed effects on malaria treatment quality [23]. In ADDO districts, the percentage of stores stocking the first line antimalarial (then sulfadoxine-pyrimethamine (SP)) increased by 15 percentage points, and the percentage of antimalarial medicines dispensed according to national treatment guidelines by 26 percentage points, while both indicators changed little in non-ADDO districts. However, the percentage of simulated malaria clients advised how to take medications fell in ADDO districts while remaining constant in non-ADDO districts. At endline there was no difference in the price of the first line antimalarial between ADDO and non-ADDO districts [23]. An independent study in 2011 compared three ADDO districts in Morogoro region with one non-ADDO district in neighbouring Pwani region, using simulated clients. They found no difference between ADDO and non-ADDO districts in a wide variety of inappropriate behaviours around antibiotic provision, including readiness to administer injections and dispense under-doses, with ADDOs actually more likely to dispense antibiotics without a prescription than non-accredited shops [10, 24]. Notably the authors found that 40% of dispensers trained under the ADDO programme no longer worked at the ADDO, with indications that they had frequently been replaced with untrained staff.

In 2011 a nationally representative survey of antimalarial providers was conducted throughout mainland Tanzania, to provide endline data for the Independent Evaluation of the Affordable Medicines Facility - malaria (AMFm), a multi-national antimalarial subsidy programme [15]. As at this time the ADDO roll out was partially complete, covering 14 out of the 21 regions, the survey provides the opportunity to compare performance of drug retailers in many regions with and without the accreditation programme in terms of their malaria-related practices. The survey provides data on a range of malaria-related indicators, including antimalarial availability, price and sales volumes, and malaria-related knowledge of outlet staff. Results from this survey indicated drug stores, including DLDB and ADDOs, accounted for 90% of all outlets stocking an antimalarial in 2011, while 3% were pharmacies and 7% general retailers.

In mainland Tanzania the artemisinin based combination therapy (ACT) artemether lumefantrine (Alu) has been the first line treatment for uncomplicated malaria since 2006, with quinine as second line. In 2011 ACT use was still quite low, with only 33% of children under five with a febrile illness obtaining an ACT [25]. Many people still took non-artemisinin therapies such as SP, especially in the private sector where they were a much cheaper alternative to ACTs. Officially Alu and quinine were POM and could be sold in pharmacies and ADDOs, but not DLDB. SP and amodiaquine could be bought OTC in any drug retailer, while all other antimalarials were POM and officially restricted to pharmacies [13]. Quinine dihydrochloride injectables were allowed in ADDOs but not DLDB, while all other injectables were prohibited in both. Oral artemisinin monotherapies were banned in Tanzania in 2008, reflecting concern that their use may contribute to artemisinin resistance (non-oral artemisinin monotherapies were still permitted for treatment of severe malaria) [26]. Malaria diagnostic tests (microscopy and rapid diagnostic tests (RDTs)) were not permitted in any drug stores.

The AMFm was implemented at national scale in Tanzania and six other countries from 2010 onwards. Hosted by the Global Fund to Fight AIDS, Tuberculosis and Malaria, AMFm aimed to increase availability, affordability and use of quality-assured ACTs, and crowd out oral artemisinin monotherapies from the market [27]. Quality-assured ACTs are ACTs that comply with the Global Fund’s quality assurance policy [28]. Subsidised ACTs were distributed through the public and private sector, including through ADDOs and DLDB, with supporting training and communications interventions. All subsidised ACTs had a green leaf logo on the packet and had a recommended retail price (RRP) of 1,000TSh ($0.64) for an adult dose in mainland Tanzania. Supporting interventions such as national level communication campaigns and local level community training were implemented alongside the main intervention. Television, newspaper and radio campaigns were conducted throughout the country, while community level dissemination was limited to two districts in each of 12 selected regions. The Independent Evaluation showed that AMFm increased access to ACTs in the private drug retail sector, with the availability of quality-assured ACTs increasing from 10% to 66% in this sector between 2010 and 2011 [8].

In this paper we explore variation in drug retailer performance across ADDO and non-ADDO regions following AMFm implementation, comparing staff knowledge of the first line drug and ACT dosing; whether outlets stocked recommended and non-recommended malaria-related products; antimalarial retail prices and mark-ups; and the market share of recommended antimalarials. The data collected provide a unique opportunity to add to the limited evidence on the effects of drug store accreditation, and represent the first study on a national scale.

## Methods

A nationally representative outlet survey was conducted between October 2011 and January 2012 [15]. Data were collected as part of the AMFm Independent Evaluation, with the sample size determined by the needs of the Independent Evaluation (full details available in Tougher et al.) [12]. Forty-nine wards were randomly selected using probability proportional to population size, stratified by urban/rural location. Wards were designated as urban or rural using National Bureau of Statistics Census classifications, with mixed wards classified as urban if more than 70% of the ward was classified as urban. We selected 20 urban and 29 rural wards, and in each selected ward all public and private outlets with the potential to stock antimalarials were visited, though only data on ADDOs and DLDB are presented in this paper. In large wards with a population over 30,000 people, wards were segmented, and one or more segments of the ward were randomly selected for the survey.

Screening criteria were used to identify outlets with an antimalarial in stock at the time of visit or within the previous 3 months. Following verbal consent, a questionnaire was conducted with the most senior staff member present, in Kiswahili, with data collected using Personal Digital Assistants (PDAs). Questions about outlet characteristics were asked, and details about every antimalarial in stock at the time of visit were recorded, as well as the volumes of each product sold in the past 7 days. Data collectors were grouped into teams of a supervisor, quality control officer and four interviewers, who all underwent two-weeks training. The quality control officer was responsible for ensuring that all eligible outlets had been visited, and revisiting a random sample of outlets to conduct spot-checks and ensure that all antimalarials had been recorded correctly.

For the purposes of this paper antimalarials were classified into four categories: (i) Alu, the first line drug in mainland Tanzania; (ii) other ACTs; (iii) artemisinin monotherapies, including oral and non-oral forms; and (iv) non-artemisinin therapies such as SP, quinine, chloroquine, mefloquine and amodiaquine. For some analysis Alu was further divided into those with the green leaf logo (i.e. AMFm subsidised drugs) and those without, and injectables were divided into ADDO-certified injectables, i.e. those permitted to be sold in ADDOs but not non-accredited drug stores, and non ADDO-certified injectables, i.e. those forbidden to be sold in all drug stores. Price data are presented separately for antimalarials in tablet and non-tablet form (including syrups, suspensions, granules and injectables), as non-tablet antimalarials have a different price distribution and tend to be more expensive than tablets, so stratifying by dosage type ensures prices and mark-ups are comparable across antimalarial categories. Price and market share data were calculated using Adult Equivalent Treatment Doses (AETDs), the amount of a drug needed to treat a 60 kg adult [29]. For example, to calculate the price per AETD of a paediatric package of Alu with 6 standard tablets (20 mg artemether and 120 mg lumefantrine), the price would be multiplied by 4 to calculate the cost of an adult equivalent dose of 24 tablets. Market share was calculated separately for ADDO and non-ADDO regions by dividing the number of AETDs of a particular antimalarial category sold by the total number of AETDs of all antimalarials sold.

Drug stores were categorised into two groups depending on their location: ADDO regions and non-ADDO regions. Drug store classification was based on region, rather than the official status of individual drug stores, as the ADDO intervention was implemented at a regional scale, and DLDB were officially not permitted in ADDO regions. In practice some drug stores operating in ADDO regions have not undergone the official ADDO upgrading or the staff who participated in this have moved on, but it is not always possible to identify these stores. Therefore we use the term “drug stores” to refer to both ADDOs and DLDBs, which are then classified by their location.

Dar es Salaam, Tanzania’s largest city and commercial centre, was a non-ADDO region at the time of data collection, but we excluded this region from the analysis as the nature of its retail drug market is very different from the rest of the country, as illustrated in the comparison of the characteristics of ADDO regions, non-ADDO regions and Dar es Salaam in Table 1. ADDO and non-ADDO regions were similar in terms of the percentage of households surveyed in in urban areas, the percentage of households classified in the poorest two socio-economic quintiles, and the percentage living within 10 min of a water source, though people in ADDO regions were significantly more likely to live within 2 km of a health facility [25]. Dar es Salaam’s characteristics were very different, with the vast majority of the population being urban and 0% of households surveyed classified in the bottom two socio-economic quintiles. Including Dar es Salaam in the analysis would therefore have compromised the comparability of the two groups.

**Table 1:** Comparison of the characteristics of the total population by area^a^

	ADDO regions	Non-ADDO regions (excluding Dar es Salaam)	Dar es Salaam
Percentage of households surveyed who:			
Live in urban areas^b^	18.8	16.5	95.8
Are in the lowest 2 socio-economic quintiles^c^	45.1 (40.9–49.3)	43.5 (38.4–48.7)	0.0
Live within 2 km of a health facility^c^	37.5 (32.7–42.5)	22.3 (17.7–27.7)	42.1 (24.3–62.3)
Live within 10 min of a water source^c^	34.6 (30.9–38.6)	33.5 (28.6–38.7)	77.4 (69.1–83.9)

^a^Includes entire population of these regions (not restricted to sampled wards)

^b^National Bureau of Statistics Census classifications, 2012

^c^Tanzania HIV/AIDS and Malaria Indicator Survey, 2011 [25]

Data analysis was performed using Stata v11, and Stata survey procedures were used to account for the survey design and stratification. Differences in availability and knowledge were assessed using the F-based design test, and differences in price by the Wilcoxon rank sum test. 2011 prices were converted to 2010 prices using the Tanzania consumer price index, and were converted to US$ using the average interbank rate for 2010 (www.oanda.com) as is standard practice for the AMFm Independent Evaluation [12].

Key informant interviews were conducted in December 2011 at national, regional and district level to gather information about AMFm implementation, other malaria-related interventions, and any other relevant contextual factors that could have impacted on malaria treatment outcomes during the study period. A total of 26 interviews were conducted with 37 people from government bodies (e.g. National Malaria Control Programme, Tanzania Food and Drug Authority, regional and district health authorities) and non-governmental organization (e.g. Clinton Health Access Initiative) [30]. Interviews followed a semi-structured interview guide, and were recorded when permission was granted, with notes also taken during the interview. The information gathered was organized by thematic codes, and the findings across interviews synthesized in a report [15]. We draw on information from these interviews in the discussion section of the paper.

Ethical approval was obtained from the Tanzanian National Institute for Medical Research, the Institutional Review Board of the Ifakara Health Institute, the London School of Hygiene and Tropical Medicine Research Ethics Committee, and the Institutional Review Board of ICF International.

## Results

We visited 148 drug stores in ADDO regions and 127 in non-ADDO regions (Table 2), of which 133 and 119 respectively were interviewed. 1833 antimalarials were recorded in these outlets.

**Table 2:** Description of drug stores visited by area

	ADDO regions	Non-ADDO regions	Total
Number of drug stores:			
Enumerated^a^	148	127	275
Screened	134	119	253
Which met the screening criteria^b^	133	119	252
With an antimalarial in stock on the day of visit	133	118	251
Number interviewed	133	119	252
Number of antimalarials recorded in drug stores	906	927	1833

^a^Outlets where at a minimum basic descriptive information was collected

^b^Outlets that had antimalarials in stock on the day of the survey or had stocked antimalarials in the past 3 months

Table 3 shows the characteristics of staff employed by the drug stores at the time of the survey (including all staff, not only those working at the time of data collection). The percentage of drug stores with at least one member of staff who had completed secondary school was 87.8% in ADDO regions and 89.3% in non-ADDO regions (*p* = 0.75). Over 96% of drug stores in both areas had a staff member with a health-related qualification. Nurse assistants, who generally have just 1 year of training, were the most common cadre of health workers in both areas, present at 64.2% of drug stores in ADDO regions and 59.1% in non-ADDO regions. There were no substantial differences in staffing between regions for any health-related cadres.

**Table 3:** Characteristics of staff employed at the time of the survey of drug stores interviewed

	ADDO regions (*N* = 133)	Non-ADDO regions (*N* = 119)
Number of staff who usually dispense drugs (median and interquartile range)	1 [1, 2]	1 [1, 2]
Percentage (95% CI) of outlets with at least one^a^:		
Person with a health-related qualification	96.1 (89.1–98.7)	96.2 (86.6–99.0)
Pharmacist	5.6 (2.2–13.6)	4.7 (1.8–11.6)
Pharmacy technician	6.1 (3.2–11.2)	5.9 (2.5–13.2)
Medical doctor	13.0 (8.3–19.9)	7.8 (3.8–15.4)
Assistant medical officer/Clinical officer/Assistant clinical officer	17.7 (12.0–25.4)	11.2 (7.1–17.2)
Nurse/midwife	7.2 (3.2–15.3)	13.3 (7.2–23.3)
Nurse assistant	64.2 (54.6–72.9)	59.1 (49.7–67.8)
Person who finished secondary school	87.8 (77.7–93.7)	89.3 (82.4–93.7)

*Denotes *p*-value < 0.05

^a^Including the owner

Knowledge of the first line antimalarial was higher among staff in ADDO regions than in non-ADDO regions (99.5% and 91.4% respectively, *p* = 0.001) (Table 4). Over 96% of staff in drug stores stocking quality-assured ACTs could state the correct dose of an ACT in adults,^1^ and over 90% in children, with no difference between areas in knowledge.

**Table 4:** Knowledge of drug store staff on the first line antimalarial and ACT dosing recommendations

	ADDO regions		Non-ADDO regions	
	N	% (95% CI)	N	% (95% CI)
Could correctly state the first line antimalarial	133	99.5 (96.3–99.9)	119	91.5 (77.3–97.1)*
Could correctly state the dosing of a quality-assured ACT in adults^a^	87	98.2 (92.1–99.6)	75	96.3 (89.6–98.7)
Could correctly state the dosing of a quality-assured ACT in children^a^	78	90.5 (75.4–96.7)	69	95.0 (87.3–98.1)

*Denotes *p*-value < 0.05

^a^Outlet staff were asked about the correct dosing of a quality-assured ACT if they had at least one quality-assured ACT in stock on the day of visit

**Fig. 1:**
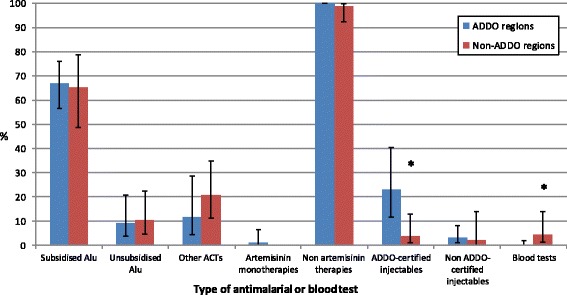
Availability of antimalarials and blood tests in drug stores by area. *: *p* < 0.05. Whiskers denote 95% confidence intervals. Blood test includes microscopy or rapid diagnostic test

AMFm subsidised Alu was stocked by 67.0% of drug stores in ADDO regions and 65.2% in non-ADDO regions (Fig. 1). There was no difference between the two areas in the availability of any type of Alu (68.2% in ADDO regions and 65.2% in non-ADDO regions, *p* = 0.74), or of other ACTs (11.9% and 20.7%, *p* = 0.32). In both areas artemisinin monotherapy was available at less than 3% of drug stores, while by contrast nearly 100% had a non-artemisinin therapy.

There was a substantial difference in the proportion of drug stores that stocked an injectable antimalarial between ADDO and non-ADDO regions (25.0% and 3.9% respectively, *p* = 0.003). The majority of these were the quinine injectable that ADDOs are permitted to stock, with 23.0% and 3.9% of drug stores in ADDO and non-ADDO regions stocking ADDO-certified injectables (*p* = 0.005) (Fig. 1). Non ADDO-certified injectables were available in under 5% of drug stores in both areas. Availability of malaria diagnostics was lower in ADDO regions (0.3%) than in non-ADDO regions (4.5%) (*p* = 0.003).

In outlets which did not stock a quality-assured ACT, staff were asked why they did not provide these products. 26.1% in ADDO regions and 24.0% in non-ADDO regions said they were “temporarily out of stock”. The next two most common responses in both areas were that “customers don’t ask for them” (20.6% in ADDO regions, 25.3% in non-ADDO regions), and that they were “too expensive” (25.4% in ADDO regions, 17.3% in non-ADDO regions). There was no difference in the proportion of people who stated regulatory reasons for not stocking between ADDO and non-ADDO regions, with 16.4% of staff in ADDO regions and 17.2% non-ADDO regions saying that the outlet was not allowed to sell them.

The median cost of subsidised Alu was slightly cheaper in ADDO regions, at $0.83 per AETD in drug stores in ADDO regions compared with $0.94 in non-ADDO regions (p = 0.005) (Table 5). The same pattern was seen with unsubsidised Alu tablets, which had a median price of $1.00 per AETD in ADDO regions and $1.25 in non-ADDO regions (*p* = 0.04). By contrast, the median price of non-artemisinin therapy tablets per AETD was higher in ADDO regions ($0.94 compared with $0.75 in non-ADDO regions). There was no significant difference in the percentage mark-up for any drug category between ADDO and non-ADDO regions, except for unsubsidized Alu tablets, for which the mark-up in ADDO regions was nearly double that in non-ADDO regions (100.0% and 53.1% respectively, *p* < 0.05) (Table 6).

**Table 5:** Retail price of antimalarials per adult equivalent treatment dose in drug stores by area^a^

	ADDO regions		Non-ADDO regions	
	N	Median Price [IQR]	N	Median Price [IQR]
Artemether lumefantrine				
Subsidised^b^ tablets	180	0.83 [0.62–1.25]	150	0.94 [0.83-1.25]*
Unsubsidised tablets	15	1.00 [0.62-1.25]	7	1.25 [1.00-6.25]*
Unsubsidised non-tablets	5	16.66 [15.00-16.66]	14	16.66 [15.00 – 16.66]
Other ACTs				
Tablets	48	6.25 [3.12-12.50]	87	6.25 [3.12-11.52]
Non-tablets	0	–	2	28.75 [20.00-37.50]
Artemisinin monotherapies				
Non-tablets^c^	1	22.50	0	–
Non-artemisinin therapies				
Tablets	387	0.94 [0.62-1.31]	376	0.75 [0.56-0.94]
Non-tablets	268	3.57 [2.81-21.97]	289	2.81 [1.87-16.48]*

*denotes *p* < 0.05

^a^Prices given in 2010 USD equivalent

^b^Artemeter lumefantrine was identified as subsidised if it bore the AMFm green leaf logo on its packaging

^c^No oral artemisinin monotherapies were recorded in drug stores

**Table 6:** Percentage retail mark-up on antimalarials in drug stores by area

	ADDO regions		Non-ADDO regions	
	N	Median Mark-up [IQR]	N	Median Mark-up [IQR]
Artemether lumefantrine				
Subsidised^a^ tablets	75	60.0 [33.3–78.6]	90	66.7 [42.9–87.5]
Unsubsidised tablets	6	100.0 [66.7–102.5]	5	53.1 [50.0–66.7]*
Unsubsidised non-tablets	3	28.20 [20.0–47.1]	6	47.06 [33.3–50.0]
Other ACTs				
Tablets	22	42.9 [32.2–53.8]	45	33.3 [− 61.1–66.7]
Non-tablets	0	–	1	11.1
Artemisinin monotherapies				
Non-tablets^b^	1	50.0	0	–
Non-artemisinin therapies				
Tablets	141	81.8 [28.6–100.0]	167	87.5 [33.3–110.5]
Non-tablets	112	66.7 [42.9–87.5]	136	50.0 [33.3–81.8]

*denotes *p* < 0.05

^a^Artemether lumefantrine was identified as subsidised if it bore the AMFm green leaf logo on its packaging

^b^No oral artemisinin monotherapies were recorded in drug stores

**Fig. 2:**
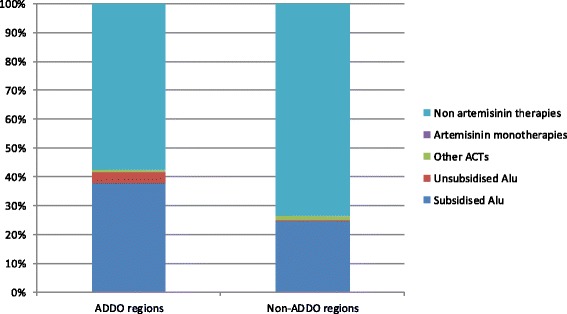
Antimalarial market share - percentage breakdown of antimalarials sold/distributed in drug stores by area^1^.*: *p* < 0.05. ^1^Antimalarials sold/ distributed in the week preceding the survey visit were included in this analysis

The market share of subsidised Alu was 38.0% in ADDO regions and 24.7% in non-ADDO regions (*p* = 0.10) (Fig. 2), with the market share of all types of Alu combined only slightly higher at 41.6% and 25.1% (*p* = 0.07) respectively. The market share of non-artemisinin therapies was higher in non-ADDO regions (73.3%) than ADDO regions (57.3%) (*p* = 0.08). For artemisinin monotherapies the market share was below 0.1% in both areas.

## Discussion

Since these data were collected the ADDO programme has been rolled out nationwide in Tanzania, and the model has been adopted or adapted in several other countries, including Ghana, Uganda and Liberia [16]. It is often cited as an important model for improving retail sector drug provision, with frequent suggestions that it can be used as a platform for expanding access to other public health commodities and services, such as family planning and integrated fever management [31]. However, the model is also acknowledged to face a number of challenges, including difficulties in providing consistent supervision and oversight for a large and growing number of ADDOs, and in maintaining a trained cadre of staff given the high turnover among dispensers [13, 24]. Evaluation of the programme’s performance is therefore very important. This paper compares practices in drug stores in regions that had undergone drug store accreditation with those that had yet to be accredited to assess how the ADDO programme affected antimalarial dispensing practices in drug stores.

In common with the two previous studies comparing ADDO and non-ADDO areas [23, 24], we find a mixed picture with ADDOs performing better on some dimensions though not on others. Before discussing these findings we note a number of study limitations. Firstly, classification of drug stores was based on region, not individual outlet. The ADDO roll out was implemented by region, so all drug stores in ADDO regions should have been ADDOs and vice versa, but sometimes this was not the case in practice. ADDOs could have moved to non-ADDO regions after being accredited, and some drug stores in ADDO regions were not accredited, for example if the drug store opened after upgrading had taken place in that region, or the outlet staff had been unable to attend ADDO training. However, as the ADDO roll out was considered to be implemented on a region by region (rather than outlet by outlet) basis, we felt that this approach to drug store classification was appropriate.

Secondly, the selection of regions that had received ADDO roll out at the time of the study was not random, but rather depended on the choices of Tanzanian Government and their partners. This raises the concern that there could have been other differences between ADDO and non-ADDO regions apart from ADDO roll out. It is reassuring to note the similarity of the two areas on key demographic indicators (Table 1), but differences could also have arisen due to variation in implementation of AMFm or other malaria-related interventions, or due to other contextual factors. To assess this we drew on key informant interviews at national, regional and district level to identify other possible factors that could have affected our results [30]. The interviews indicated that implementation of AMFm supporting interventions was relatively uniform across the country, as it relied primarily on mass media. Local level communications activities were held in selected districts only, but these were very small scale, and held in half of the ADDO regions and half the non-ADDO regions, so are unlikely to have affected the relative performance of these areas in our findings. A separate USAID-funded malaria communications and training campaign was conducted at the time, but again the target districts were evenly spread between ADDO and non-ADDO regions. The key informant interviews did not identify any other interventions or contextual factors likely to have affected our indicators.

A third limitation is that results were based on reported data from outlet staff, and may have been subject to recall bias, or staff may have biased their answers if they were worried about acting outside the regulations. For example, staff may not have declared POMs they were not allowed to sell, which could particularly have led to under-reporting of ACT availability in non-ADDO regions where drug stores were not allowed to stock these POMs. Staff may also have understated their retail price for subsidised ACTs if they were charging more than the RRP. Finally, this study is based on the range of performance measures available in the outlet survey related to antimarial provision and malaria-related knowledge. Other indicators such as antimalarial dispensing practices and appropriate referrals have not been captured, and non-malarial products such as antibiotics have not been included.

### Dimensions of performance which were similar in ADDO and non-ADDO regions

Drug stores in ADDO regions and non-ADDO regions performed similarly on a number of indicators. For example, the percentage of drug stores with a staff member with a health-related qualification was very high in both areas, although by far the most common cadre of staff in both areas was nurse assistant, which requires just 1 year of training. Knowledge of dosing recommendations was similar in both areas, with over 90% of staff stocking quality-assured ACTs able to state their dosing recommendations correctly in all areas.

There was also no difference in availability of any antimalarial drug categories between drug stores in ADDO regions and non-ADDO regions, apart from ADDO-certified injectables which were more common in ADDO regions (discussed below). Drug stores in non-ADDO regions were not permitted to stock Alu or other ACTs, so their high availability could indicate a lack of supervision of drug shops in these regions. However, the regulatory authorities in Tanzania have reported that they chose not to enforce the regulation restricting Alu sales strongly in rural areas, due to lack of alternative legitimate private providers and frequent public sector stock outs [3, 15]. Indeed there was no difference in the proportion of drug store interviewees who stated regulatory reasons for not stocking quality-assured ACTs between ADDO and non-ADDO regions.

Availability of non-artemisinin therapies was very high in ADDO and non-ADDO regions, with the most commonly sold antimalarial in this category being SP, an OTC medication. Given SP’s previous status as first line drug, its relatively low price, and the fact that both ADDOs and non-ADDOs are permitted to sell such OTCs, it was not surprising to see high availability and market share of non-artemisinin therapies in both areas. In contrast, artemisinin monotherapies were banned in both drug store types, and availability and market share was minimal in both areas.

### Dimensions of performance which were better in ADDO regions

Despite these similarities, drug stores in ADDO regions performed better than those in non-ADDO regions in some respects. Although there was no difference in Alu availability between the two areas, there was weak evidence to show that drug stores in ADDO regions had a higher Alu market share than those in non-ADDO regions, while the reverse was true for non-artemisinin therapies. This might be explained by several factors. First, it could have reflected lower Alu prices; while the median Alu price exceeded the RRP in both ADDO and non-ADDO regions, it was closer to the RRP in ADDO regions, with a median $0.11 less per AETD than in non-ADDO regions. Higher Alu market share could also partly reflect the better knowledge of the first line antimalarial in ADDO regions, though knowledge was above 90% in all areas. Staff in ADDOs may have felt a greater sense of responsibility and confidence to provide advice about the recommended antimalarial to their customers due to the training they received as part of the ADDO upgrading programme. It could also have reflected the greater proximity of the population in ADDO regions to health facilities (Table 1) which may have facilitated communication on the first line antimalarial to community members.

The availability of non ADDO-certified injectables was low in all areas, while the availability of ADDO-certified injectable antimalarials was very low in non-ADDO regions and significantly higher in ADDO regions. This was mainly due to the presence of quinine dihydrochloride injectables, which are permitted in ADDOs but not in unaccredited drug stores. In contrast, a higher proportion of outlets had blood testing available in non-ADDO regions; as blood tests were not permitted in either drug store type, this could suggest the need for stricter regulations and supervision in this area. However, in response to data that show that the majority of people purchasing ACTs in drug stores are not infected with malaria [32], the Tanzanian government and partners have piloted the promotion of rapid diagnostic tests for malaria in drug stores, so the regulatory status of blood testing may be reviewed [33].

## Conclusion

The ADDO model has now been rolled out nationwide in Tanzania and emulated in a number of other countries. This study showed that performance on some malaria-related indicators was better in ADDO regions than in other areas. However, these differences were relatively small, and there was no difference on several other measures of performance. Even where ADDO regions did perform somewhat better than non-ADDO regions, there was still considerable room for improvement, with 31% of drug stores in ADDO regions not stocking the first line antimalarial, and non-artemisinin therapies continuing to account for nearly 60% of the antimalarial market, despite ACT subsidies implemented under AMFm.

On the basis of these results alone one would be cautious about promoting the ADDO model, although we recognize that this paper’s results cover a limited range of malaria-related indicators, accounting for only one area of ADDO performance. Unfortunately other literature comparing ADDOs with non-ADDO drug stores is limited and inconsistent, comprising one positive internal evaluation of the pilot programme [23], and one more negative independent evaluation [24]. Thus, while these results contribute to the literature in this area, to make evidence-based decisions on drug shop policy, it will be essential that robust evaluations of any future ADDO-style programmes are conducted, to assess their performance across a wide range of indicators, and to explore their cost-effectiveness compared to alternative approaches to improve case management in the community.

## Abbreviations

ACT: Artemisinin based combination therapy
ADDO: Accredited Drug Dispensing Outlet
Alu: Artemether Lumefantrine
AMFm: Affordable Medicines Facility – malaria
DLDB: Duka la dawa baridi
IMCI: Integrated management of childhood illnesses
OTC: Over-the-counter
POM: Prescription only medicine
RRP: Recommended retail price
SP: sulfadoxine pyrimethamine

## Footnotes

1 Dosing knowledge was only tested in outlets stocking quality-assured ACTs. Dosing was categorized as correct if the interviewee could correctly state the state the number of tablets to be taken, the number of times per day and over how many days the tablets should be taken.
